# Liver Transplantation for Autoimmune Hepatitis: 20 Years of Tertiary Centre Experience

**DOI:** 10.5152/tjg.2024.24464

**Published:** 2024-12-16

**Authors:** Osman Sağlam, Muhsin Murat Muhip Harputluoğlu, Yılmaz Bilgiç, Sezai Yılmaz, Fatma Hilal Yağın, Cumali Efe

**Affiliations:** 1 Department of Gastroenterology, Afyonkarahisar Health Sciences University, Afyonkarahisar, Türkiye; 2 Department of Gastroenterology, İnonu University Faculty of Medicine, Malatya, Türkiye; 3 Department of Gastroenterology, İstanbul Medipol University Faculty of Medicine, İstanbul, Türkiye; 4 Liver Transplant Institute, İnonu University, Malatya, Türkiye; 5 Department of Biostatistics and Medical Informatics, İnonu University Faculty of Medicine, Malatya, Türkiye; 6 Department of Gastroenterology, Harran University Faculty of Medicine, Şanlıurfa, Türkiye

**Keywords:** Autoimmune hepatitis, liver transplantation, post-transplant complications

## Abstract

**Background/Aims:**

We analyzed the frequency of complications and survival rates in patients with autoimmune hepatitis (AIH) who underwent liver transplantation at a high-volume transplant center.

**Materials and Methods:**

Patients who underwent transplantation for AIH at the İnönü University Liver Transplantation Institute between January 2002 and December 2021 were included. Patients with a confirmed diagnosis of AIH, without concomitant chronic liver disease, were included in the study.

**Results:**

We included 51 patients (31 female) with a median age of 38.5 years (18-65 years). The 12-month and 60-month survival rates were 86.3% and 80.9%, respectively. During a median 2.22 years follow-up, 9 patients died. Six patients died due to systemic infection, 1 due to biliary complications, and 2 patients due to graft rejection. Autoimmune hepatitis recurrence developed in 6 (11%) patients. Overall, biliary complications developed in 56% (28/51) of patients following liver transplantation, and graft rejection occurred in 22% (11/51) of patients.

**Conclusion:**

Our results suggest that the outcome of AIH following liver transplantation is good, with a survival rate of up to 80%. Post-transplant biliary complications are common; therefore, close follow-up is necessary.

**Main Points**

- Liver transplantation is a life-saving treatment for acute and chronic liver failure due to autoimmune hepatitis, with survival rates of up to 80%.
- The main cause of death after transplantation is infection.
- The incidence of biliary complications may increase in patients undergoing liver transplantation for autoimmune hepatitis.

## Introduction

Autoimmune hepatitis (AIH) is a multifactorial liver disease of unknown origin characterized by chronic immune-mediated liver disease with elevated aminotransferase and immunoglobulin G (IgG) levels, the presence of circulating autoantibodies, interface hepatitis on histologic examination, and a favorable response to immunosuppression. If left untreated, AIH can progress to end-stage liver disease and cirrhosis. Liver transplantation (LT) is the ultimate therapeutic option for patients with AIH and end-stage liver disease.^1,2^

According to the European Liver Transplant Registry (ELTR) records between 1988 and 2016, 2% of all patients who underwent LT and 5% of those who underwent LT for cirrhotic indications had AIH. This study suggested that LT is a life-saving intervention for AIH patients, with 1-year and 5-year survival rates ranging from approximately 56% to 100% and 73% to 86%, respectively.^3^ However, AIH recurrence (rAIH) has been reported in 15%-40% of patients.^4^ There are unknown issues regarding the long-term follow-up of AIH patients who underwent LT. This study aimed to analyze the long-term outcomes of patients with liver transplantation for AIH (AIH-LT) from a high-volume LT center.

## Materials and Methods

### Data Collection

Data were obtained from an online hospital medical record database that contained records of all liver transplants performed at İnönü University between January 2002 and December 2021. Patients who underwent transplantation for AIH were selected as the focus of our study.

### Inclusion and Exclusion Criteria

**Figure 1.**
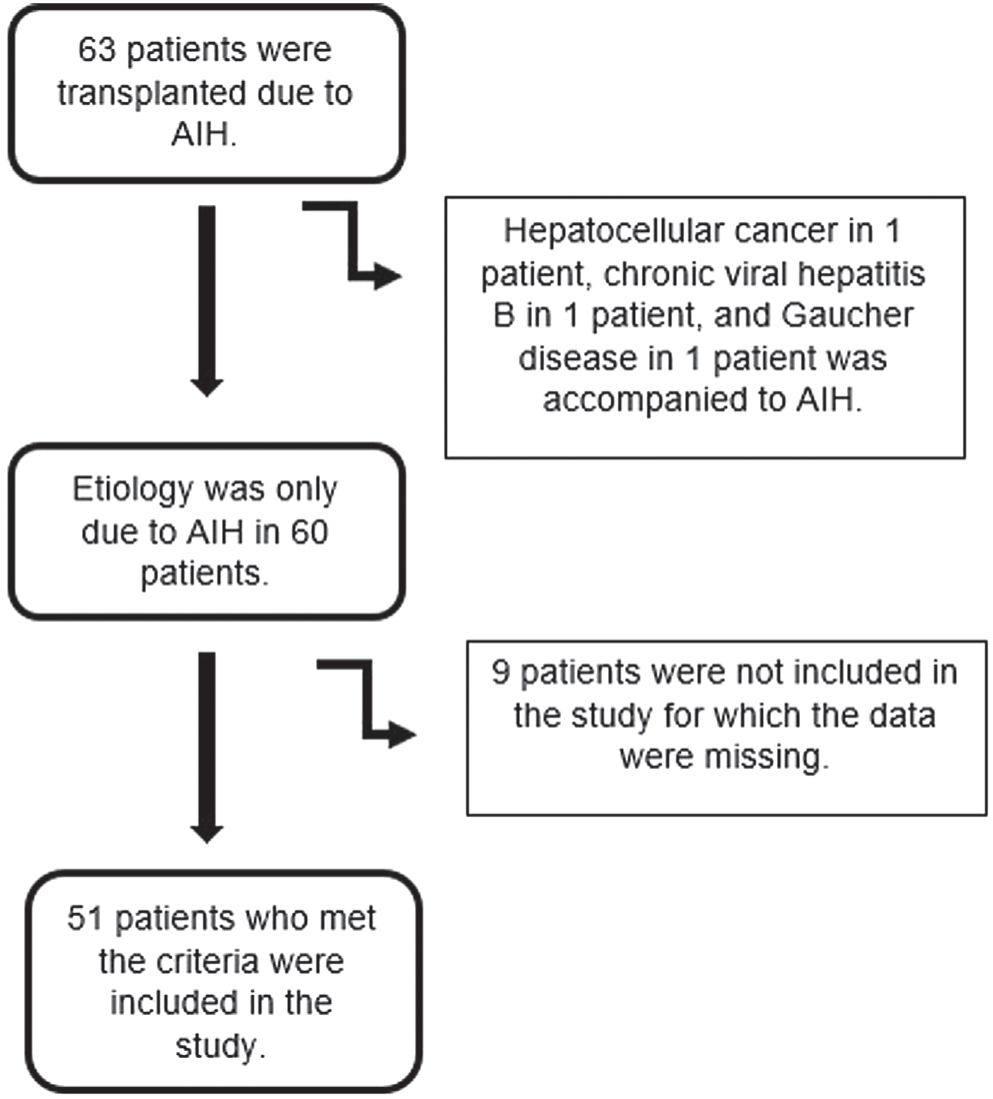
Patient selection algorithm.

Patients with incomplete data and those lost to follow-up were excluded from the study. Autoimmune hepatitis patients with features of primary biliary cirrhosis (PBC) or primary sclerosing cholangitis (PSC) overlap syndrome, and hepatocellular cancer, chronic viral hepatitis B, and Gaucher disease were also excluded from the study (Figure 1).

### Assessments and Selection of Immunosuppressive Treatment

Rejection or rAIH was determined based on liver biochemistry, imaging, and histological findings. Data on patient age at the time of LT, sex, transplantation type, immunosuppressive treatment, survival duration, rejection, biliary complications, and rAIH were used for the study. Post-transplant immunosuppressive drug selection is individualized according to patient characteristics.

### Ethics Committee Approval

The study was approved by the Ethics Committee of İnönü University (approval number: 2022/3139; date: March 08, 2022) and the study was performed according to the principles stated in the Declaration of Helsinki. Due to the retrospective nature of the study, informed consent was not obtained.

### Statistical Analysis

Categorical (qualitative) variables are expressed as numbers (percentages). Quantitative variables were summarized using median, minimum, and maximum values. Survival was analyzed using the Kaplan–Meier method, and differences in survival with the log-rank test. Prognostic factors were determined by univariate analyses using the Cox proportional hazards regression model. The follow-up period was defined as the time elapsed from transplantation until death for any reason. Statistical tests with a *P*-value less than .05 were considered significant. All statistical analyses were performed using IBM Statistical Package for Social Sciences Statistics for Windows, version 26.0 (IBM SPSS Corp.; Armonk, NY, USA).

## Results

We included 51 patients with AIH (n = 31, female) with a median age of 38.5 years (18-65) at the time of LT. Most patients (98%) had chronic hepatitis, while 1 had acute liver failure. Forty-eight patients (94%) received transplants from living donors, and the remaining 3 patients (6%) underwent cadaveric transplantation. The median follow-up period was 2.22 years. For living patients, the median follow-up period was 3.33 years, while for deceased patients, it was 2 months. The general characteristics of the study population are summarized in Table 1. A total of 42 (82%) patients were living while 9 (18%) patients died. Causes of mortality were systemic infections (pneumonia, urinary tract infection (UTI), and peritonitis) in 6 patients, post-LT biliary complications in 1 patient, and graft rejection in another 2 patients (Table 2).

Following LT, mycophenolate mofetil + tacrolimus + steroids were administered to 41.1% of the patients, and tacrolimus + steroids were administered to 15.6%. Among the patients, 43.1% used different combinations of steroids, mycophenolate mofetil, tacrolimus, and everolimus (Table 1).

**Figure 2.**
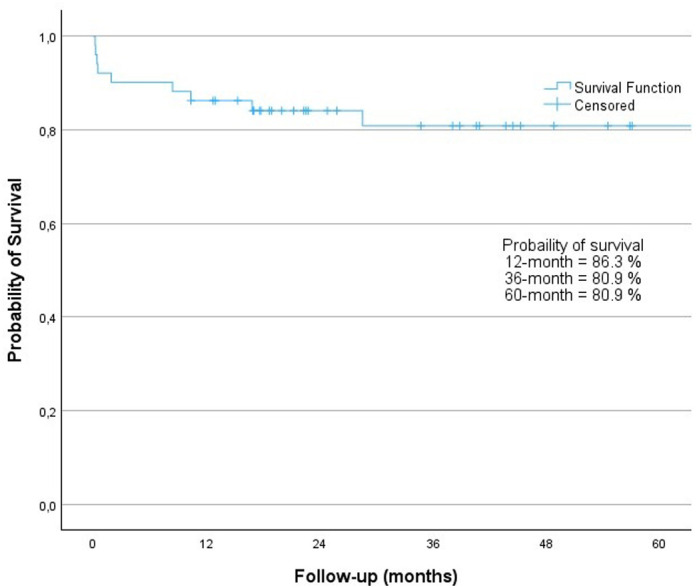
Survival of patients after liver transplantation. The 12-, 36-, and 60-month probability of survival was 86.3%, 80.9%, and 80.9%, respectively. Cumulative probabilities were calculated using the Kaplan–Meier method.

The 12-month survival rate in our study was 86.3%, while the 36-month and 60-month survival rates were 80.9% (Figure 2). Rejection occurred in 11 (21.56%) patients during the follow-up period. The median duration from LT to the development of graft rejection was 150 (7-2314) days. Recurrent autoimmune hepatitis (rAIH) developed in 6 patients (11.76%), with a median of 403 (16-2624) days after LT. Three patients (5.88%) experienced both graft rejection and rAIH.

One patient developed cirrhosis 114 months after the transplantation. One patient had re-LT 6 days later because of hepatic vein thrombosis. Biliary complications developed in 28 patients (56%). Of these, 79% were strictures, 10.5% were leaks, 7% were both strictures and leaks, and 3.5% were stones. The median development time of biliary complications was 57.5 (9-611) days following LT (Table 3).

**Figure 3.**
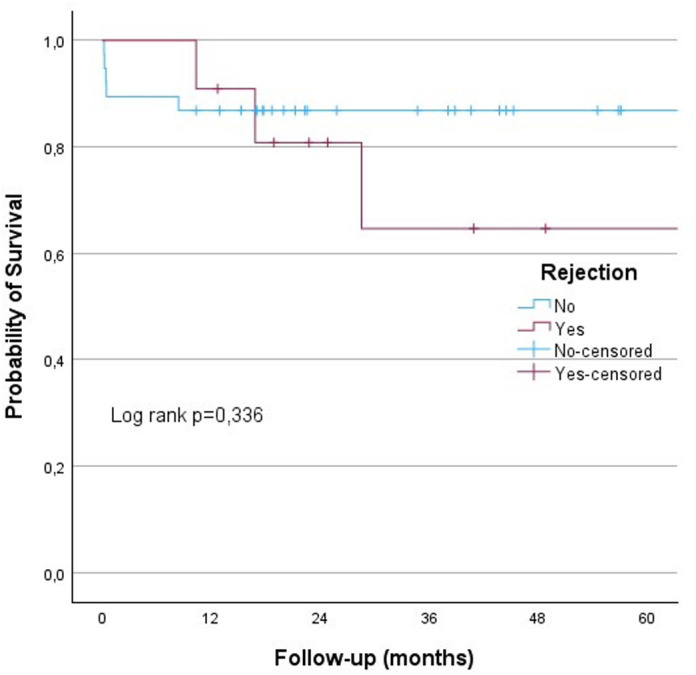
Kaplan–Meier analysis showed that probability of a survival was no different in those with rejection and without rejection (log-rank test, *P* = .336).

**Figure 4.**
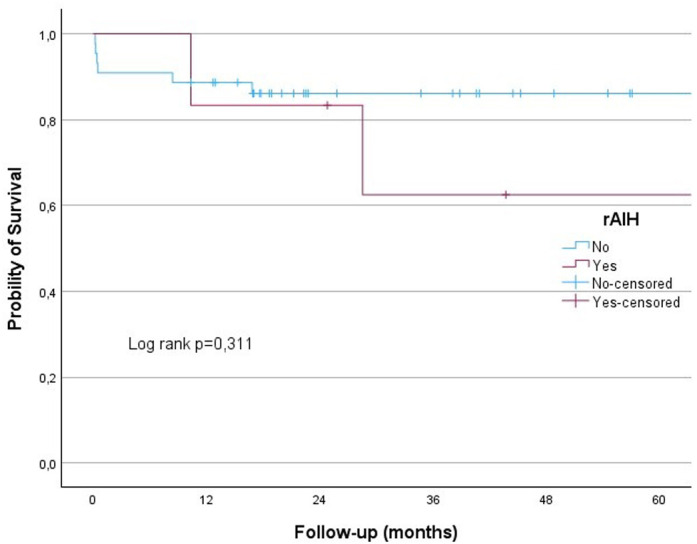
Kaplan–Meier analysis showed that probability of a survival was no different in those with rAIH and without rAIH (log-rank test, *P* = .311). rAIH, recurrent autoimmune hepatitis.

**Figure 5.**
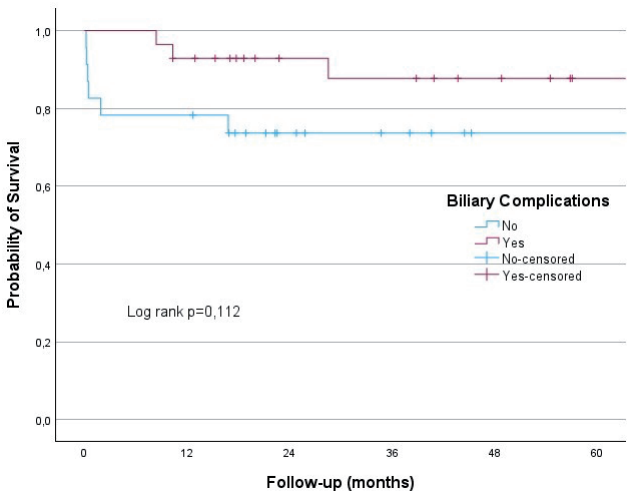
Kaplan–Meier analysis showed that probability of a survival was no different in those with biliary complications and without biliary complications (log-rank test, *P* = .112).

The results from the univariate analysis presented in Table 4 show that gender, patient age at the time of transplantation, donor age at the time of transplantation, pre-transplant MELD-Na score, rejection, rAIH, and biliary complications did not affect survival. No significant differences were observed in the 60-month survival rates for rejection, rAIH, and biliary complications (log-rank *P* = .336, *P* = .311, and *P* = .112, respectively) (Figures 3-5).

## Discussion

In this study, the 12-month survival rate for patients who underwent LT for AIH was 86.3%, while the 36-month and 60-month survival rates were 80.9%. Six patients died due to infection, 1 patient due to biliary complications, and 2 patients due to rejection. The rejection, rAIH, and biliary complication rates were 21.5%, 11.7%, and 56%, respectively. No significant differences were observed in the 60-month survival rates for rejection, rAIH, and biliary complications (log rank *P* = .336, *P* = .311 and *P* = .112, respectively) (Figures 3-5).

Between 1988 and 2016, AIH accounted for 2% of all liver transplants and 5% of transplants were performed for cirrhotic indications in the ELTR.^5^ Previous studies on patients who underwent LT for AIH reported survival rates ranging from 56% to 100% at 5 years and 73% to 86% at 10 years of follow-up. In our patient group, 12-month and 60-month survival rates were 86.3% and 80.9%, respectively. Heinemann et al^3^ reported that most deaths following AIH-LT were attributed to infections (29.5%), followed by neoplasia and graft rejection. Bacterial infections (13.8%) and pulmonary infections were the most common causes of infection-related death. In our study, 6 patients died due to infection (66.66%), 2 due to rejection (22.22%), and 1 due to biliary complications (11.11%). Four deaths were attributed to pneumonia, 1 patient had a UTI, and 1 patient had peritonitis. Considering Heinemann’s study and our study, we can conclude that infections are the most common cause of mortality following AIH-LT. In terms of survival rates, our center’s rates were quite satisfactory.

Compared to older patients (≥47 years), younger adults (16-46 years) undergoing LT for AIH have been observed to have an increased risk of mortality. Additionally, donor age ≥53 years increases the risk of mortality.^3^ Specifically, younger age at LT (≤42 years) has been associated with a higher risk of AIH recurrence, which in turn is significantly linked to both graft loss and death.^6^ In our study, the age of the deceased patients ranged from 21 to 53 years at the time of transplantation, and 3 patients were older than 47 years. The donor ages of the deceased patients ranged from 23 to 46 years (Table 2). By univariate Cox proportional hazard regression analysis, patient age at the time of transplantation (HR: 0.99, 95% CI: 0.95-1.04, *P* = .803) and donor age at the time of transplantation (HR: 1.03, 95% CI: 0.96-1.11, *P* = .351) were not associated with survival (Table 4). It is possible that the small number of patients included in our study contributed to this outcome.

After LT, AIH can develop in de novo or rAIH forms.^7^ There is evidence of overlap between autoimmune and alloimmune responses in patients with autoimmune liver diseases after LT. Although de novo AIH is reported in approximately 4%-6.6% of AIH-LT cases, the occurrence of rejection varies greatly among different centers and time periods, ranging from 9% to 82%. This variability has been attributed to differences in immunosuppressive treatment and biopsy protocols. However, it has also been suggested that this may be related to the variability in the diagnostic criteria for rejection.^8^ Situations such as the socioeconomic status and education level of patients can be determinants of patient compliance and, thus, treatment success.^9^ In a study, it was found that AIH-LT patients had a higher risk of death due to rejection than patients with alcoholic cirrhosis, PBC, or PSC.^3^ Another study examining 736 AIH-LT patients from 33 centers reported rejection in 35% of cases and a combination of recurrence and rejection in 11% of cases.^6^ In our study, rejection occurred in 11 patients (21.56%) and both rejection and rAIH developed in 3 patients (5.88%). The rejection and rAIH rates in our study appeared to be lower than those reported in the literature. Although we could not determine the exact reasons for the low rates found in our study, it is possible that they are related to the selection of immunosuppressive therapies.

The frequency of rAIH varies between centers, ranging from 0% to 69% during a 5-year follow-up period.^6^ This variability appears to be related to differences in the protocols used for liver biopsies, the small number of patients in each series, and variations in the follow-up duration in different studies.^10^ In our study, rAIH was detected in 6 patients (11.7%). Among these patients, histological examination detected the coexistence of rejection and recurrence in 3 cases. Long-term low-dose corticosteroid use (prednisolone 5-10 mg) in combination with other immunosuppressive agents appears to reduce rAIH without compromising patient and graft survival.^11^ However, the latest guidelines from the American Association for the Study of Liver Diseases (AASLD) recommend discontinuing glucocorticoids after LT and monitoring patients for rAIH.^12^ In our study, 4 patients (7.8%) were treated with steroids alone, while other patients were transitioned to other immunosuppressive therapies (Table 1) in line with the AASLD recommendations. Although the wide range of rAIH frequencies reported in the literature makes it difficult to compare our study results with those of other studies, the low recurrence rate in our study may be attributed to the use of long-term immunosuppressive treatment.

Biliary complications are the most common following LT.^13^ Multiple factors can contribute to the development of biliary complications. Anastomotic lesions are mostly caused by technical issues, whereas non-anastomotic lesions result from ischemia or immune reactions.^14^ Due to the technical challenges associated with living donor liver transplantation (LDLT), biliary complications have been reported to be more frequent in LDLT than in deceased donor transplants.^15^ The overall incidence of biliary complications after LDLT ranges from 6% to 40%. These complications are expected to occur within a few weeks or months after transplantation, usually within the first year and approximately 5-8 weeks on average.^16,17^ In a study by Chouik et al^18^, the incidence of biliary complications after AIH-LT was 25.3%. In our patient group, 48 patients (94%) underwent transplantation from living donors (Table 1). The high rate of biliary complications in our study could be attributed to factors such as donor type, ischemia time, surgical technique, and the potential negative impact of AIH on the bile ducts. Our results highlight the importance of considering and being alert to post-transplant biliary complications in patients with AIH.

In patients with AIH undergoing LDLT, there has been an observed increase in the risk of death related to biliary complications compared with deceased donor transplants.^3^ However, a meta-analysis found that LDLT was associated with a higher incidence of biliary complications than deceased donor transplants, but this did not affect overall survival.^19^ Among the 9 patients who died in our study, 1 died of biliary complications. Our results suggest that although biliary complications occur at a high frequency in patients undergoing transplantation for AIH, they do not affect survival (Table 4).

This study yielded significant results regarding the follow-up course of transplant patients with AIH, but there are certain limitations that must be acknowledged. First, it was conducted retrospectively. Second, the small number of patients in our study restricted our ability to interpret the data obtained. Finally, the fact that our research was conducted at a single center restricts the generalizability of our findings.

Liver transplantation for AIH is a life-saving treatment, with survival rates of up to 80%. Infections are the most common cause of death after AIH-LT. Our results suggest that, although biliary complications do not have an impact on survival in patients undergoing transplantation for AIH, their frequent occurrence necessitates vigilance and monitoring during follow-up.

## Figures and Tables

**Table 1.** Clinical Patient Characteristics

Age	Median: 38.5 Years (18-68)		
	Male: Median 42 Years (18-68)		Female: Median 36.5 Years (18-62)
Gender	Male: 20 (39.21%) patients		Female: 31 (60.78%) patients
Indication of transplantation	ALF: 1 patient		CLF: 50 patients
Donor type	LDLT: 48 patients		Cadaveric: 3 patients
Survival	Alive: 42 patients		Deceased: 9 patients
Follow-up	For survived patienst: median 3.33 years	For dead patients: median 2 months	For all patients: 2.22 years
Immunosuppressive therapies	**Type of Treatment**		**Frequency (n, %)**
	Mycophenolate mofetil + tacrolimus + steroid		21 (41.1)
	Tacrolimus + steroid		8 (15.6)
	Mycophenolate mofetil + tacrolimus + steroid + everolimus		7 (13.7)
	Tacrolimus + steroid + everolimus		4 (7.8)
	Steroid		4 (7.8)
	Mycophenolate mofetil + tacrolimus		2 (3.9)
	Takrolimus		1 (1.9)
	Mycophenolate mofetil + steroid + everolimus		1 (1.9)
	Mycophenolate mofetil + everolimus		1 (1.9)
	Mycophenolate mofetil + steroid		1 (1.9)
	Steroid + everolimus		1 (1.9)
	Total		51 (100)

ALF, acute liver failure; CLF, chronic liver failure; LDLT, living donor liver transplantation.

**Table 2.** Characteristics of Deceased Patients

Patient No.	1	2	3	4	5	6	7	8	9
Sex	Male	Male	Female	Female	Female	Female	Female	Female	Female
Age at the time of transplant	53	52	44	38	30	26	24	51	21
Type of transplantation	LDLT	LDLT	Cadaveric	LDLT	LDLT	LDLT	Cadaveric	LDLT	LDLT
Donor age	25	23	46	43	32	45	35	24	23
Follow-up time (days)	7	57	12	505	14	856	252	6	310
Rejection	–	–	–	+	–	+	–	–	+
rAIH	–	–	–	–	–	+	–	–	+
Biliary complication	–	–	–	–	–	Stricture+Leakage	Stricture+Leakage	–	Stricture+Leakage
Retransplatation	–	–	–	–	–	–	–	+	–
Transplantation indication	CLF	CLF	CLF	CLF	CLF	CLF	CLF	CLF	ALF
Cause of death	Peritonitis	Rejection	Pneumonia	Pneumonia	UTI	Pneumonia	Biliary complication	Pneumonia	Rejection

ALF, acute liver failure; CLF, chronic liver failure; LDLT, living donor liver transplantation; rAIH, recurrent autoimmune hepatitis; UTI, urinary tract infection.

**Table 3.** Post-Transplant Complications

Post-transplant Complications	Number of Patients (n, %)	Development Time (mean, min., max., days)
Rejection	11 (21.56)	150 (7-2314)
rAIH	6 (11.76)	403 (16-2624)
Rejection + rAIH	3 (5.88)	26 (16-173)
Cirrhosis	1 (1.96)	3437
Hepatic vein thrombosis	1 (1.96)	6
Biliary complications Stricture Leakeage Stricture + leakage Stone	28 (55) 22 (79) 3 (10.5) 2 (7) 1 (3.5)	57.5 (9-611)

min., minimum; max., maximum; rAIH, recurrent autoimmune hepatitis.

**Table 4.** Univariate Cox Regression of Prognostic Factors for Overall Mortality

	Hazard Ratio (CI 95%)	*P*
Female gender	0.44 (0.09-2.11)	.302
Patient age at the time of transplantation	0.99 (0.95-1.04)	.803
Donor age at the time of transplantation	1.03 (0.96-1.11)	.351
Rejection	0.58 (0.14-2.32)	.441
rAIH	0.52 (0.11-2.48)	.408
Biliary complications	2.93 (0.73-11.79)	.129

rAIH, recurrent autoimmune hepatitis.

## Data Availability

The data that support the findings of this study are available on request from the corresponding author.
